# Editorial: Innovations in dementia policies and services

**DOI:** 10.3389/frdem.2026.2006454

**Published:** 2026-10-13

**Authors:** Walter D. Dawson, Alison J. Canty, Joaquín Migeot, Laura O'Philbin, Sherril B. Gelmon

**Affiliations:** 1 Department of Neurology, Oregon Health & Science University, Portland, OR, United States; 2 Institute on Aging, Portland State University, Portland, OR, United States; 3 Global Brain Health Institute, University of California San Francisco, San Francisco, CA, United States; 4 Wicking Dementia Research and Education Centre, University of Tasmania, Hobart, TAS, Australia; 5 Global Brain Health Institute, Trinity College Dublin, Dublin, Ireland; 6 Latin American Brain Health Institute (BrainLat), Universidad Adolfo Ibañez, Santiago de Chile, Chile; 7 The Alzheimer Society of Ireland, Dublin, Ireland; 8 Oregon Health & Science University - Portland State University School of Public Health, Portland, OR, United States

**Keywords:** Alzheimer's disease, care and support, dementia, dementia services, health policy, innovation, technology

## 1 Introduction

There is an increasing global consensus that dementia is a significant public health issue (Chapman et al., 2006; Jackson et al., 2023; World Health Organization, 2017). As the global population ages and the number of people living with dementia increases, dementia will continue to grow in salience (Nichols, E., and GBD 2019 Dementia Forecasting Collaborators, 2022). The complexities associated with dementia require innovative approaches to the provision of care and support as well as to the design of service systems at local and national levels across the globe that can truly meet the unique needs of people living with dementia and their care partners. These systems need policies to provide the foundation for better dementia care and support.

Innovations in dementia policies and services are typically discussed and reported far less frequently in the general dementia literature than innovations in clinical care, diagnostics, treatment, or basic science. The wide variation in approaches to policy and regulatory environments, as well as unique sociocultural contexts across the world, offer multiple opportunities to showcase innovative programs, networks, and policies that make non-clinical services and supports available and accessible to people living with dementia and their care partners. Highlighting and sharing these innovations in dementia services and policies is needed to support greater accessibility.

This Research Topic of *Frontiers in Dementia*, Innovations in Dementia Policies and Services, includes nine original research manuscripts as well as two systematic reviews, two reviews, one mini review, nine perspectives, one brief research report, and two opinions, all of which advance a better understanding of the breadth of innovation taking place in dementia policies and services. Some of the thematic areas that emerged from the submissions to this Research Topic include Access for Underserved Populations, Nature-based Supports and Programs, Creative Pursuits in Dementia Services, Specific Policies, and Technological Innovations for Care and Support. In total, 26 papers with 178 co-authors are included within this edition of Frontiers in Dementia, with these co-authors listing affiliation with institutions in 23 countries across the globe.

## 2 Access for underserved populations

Inequities in access to dementia prevention, diagnosis, care, and support remain a major challenge across diverse health systems and communities (Calil et al., 2020; Giebel et al., 2025). These barriers are often amplified for populations facing social disadvantage, cultural exclusion, geographic limitations, low income, migration, or limited access to specialized services (Bernstein Sideman et al., 2022; Giebel et al., 2025; Ibrahim and Mohamed, 2025), being associated with worse brain health (Migeot et al., 2025). The articles in this theme highlight innovations designed with and for underserved populations, including community-embedded prevention models, culturally responsive care pathways, public memory clinic services, Indigenous-led adaptation of educational resources, and frontline approaches that connect local assessment with policy and practice. This set of evidence shows that improving dementia care equity requires services that are locally grounded, culturally meaningful, and responsive to the realities of the communities they aim to serve.

Bell et al. (2026) in a “Perspectives” article describes the Multimodal Intergenerational Social Contact Intervention (MISCI), a community-embedded program designed to address dementia risk factors of reduced loneliness and depressed mood through culturally meaningful intergenerational engagement.

Nair et al. extend this equity-oriented focus by proposing a Community Health and Wellbeing Worker-led model in a “Perspectives” article, designed to provide proactive, culturally responsive dementia support across the care continuum. The innovation lies in strengthening existing neighborhood-based roles, offering a scalable pathway to improve early identification, continuity, and access for underserved communities.

In an “Original Research” article from Peru, Custodio and Dorell explore how family care partners experience and manage behavioral and psychological symptoms of dementia, highlighting the emotional impact of caregiving and the importance of culturally responsive dementia supports for underserved populations in Latin America.

In an “Original Research” article, Fischer et al. illustrate how the content and delivery of a web-based educational program to increase knowledge around dementia risk factors and help create lifestyle changes were culturally inappropriate for Métis communities in Alberta, Canada. They found that in-person engagement with Métis communities is a crucial step to co-develop resources that are culturally appropriate and reflect Indigenous knowledge and worldviews.

A “Review” focused mostly on Europe and the United States (US), led by Aguzzoli et al., highlights persistent inequalities among people from ethnic minority groups. The authors describe both reduced access to primary care and lower adherence to treatments compared to majority counterparts in their communities, providing a reminder of the ongoing need for culturally tailored interventions across the globe, and within national and local communities.

In another “Original Research” article from Argentina, Solis et al. describe 10 years of experience from a public memory clinic serving older adults in a predominantly low-income region. The authors' work shows how a specialized dementia care model can be adapted to the needs of an underserved population.

DeDecker et al.'s “Perspective” describes a novel approach taken in Ontario, Canada that seeks to address the gaps in assessment and support of people living with dementia. The program embeds structured cognitive and functional assessments within community-based organizations, shares those assessment results with primary care providers, and links standardized data to a provincial registry. This addresses access issues while creating a standardized minimum dataset that can be used to inform policy and practice in real time.

## 3 Nature-based supports and programs

Nature-based programs and supports that engage people living with dementia and their care partners have been shown to offer benefits such as improved mental health, physical activity, cognitive stimulation, and social connection (Bennett et al., 2022; Murroni et al., 2021; White et al., 2018). Nonetheless, many people with dementia and their care partners are challenged to access such activities given cost, awareness, issues with mobility, environmental design, and dementia-related stigma, let alone the cognitive changes associated with dementia (Nguyen et al., 2025). The innovations highlighted in this group of articles draw upon the expertise of authors from Italy, the United Kingdom (UK), and Australia.

Thompson et al. present a “Mini-Review” of nature-based living labs, extending their application in dementia research from a focus on indoor settings, technological tools, and aged care systems to engagement in nature which has been shown to offer wide-ranging benefits to people living with dementia.

In Northern Italy, Dalla Costa et al. contributed a” Perspective” where they describe how farms can become places where people living with dementia are not only cared for but are empowered to be active participants in meaningful shared work in nature. They write that this low-cost intervention, which capitalizes on existing resources and volunteer frameworks, can be easily adapted for other agricultural and community contexts.

In a “Review,” the UK-based team of Gill et al. seek to map the existing evidence base and explore how time in nature and structured nature-based interventions may offer benefits to people living with dementia as well as their care partners. This review gathers evidence that both indoor and outdoor nature-based interventions may impact psychological and emotional health, wellbeing, and social engagement.

## 4 Creative pursuits

The World Health Organization's arts and health movement highlights the growing recognition that creative practices, including music, visual arts, storytelling and performance, can enhance physical, emotional and social wellbeing across the lifespan (World Health Organization, n.d.). This arts and health perspective is emerging as a powerful approach in the context of dementia, where cognitive changes create shifts in abilities where some domains decline but others remain intact. Creative pursuits can support identity, evoke emotional expression and strengthen relationships in ways that traditional biomedical approaches alone cannot achieve (Dowlen et al., 2018; Letrondo et al., 2023; Rusted et al., 2006). This Research Topic of articles shares examples of how integrating creative modalities into dementia care and service design can enrich daily experiences, improve communication and foster environments that uphold dignity and connection. As interest in arts-based innovation continues to grow, these approaches will be able to inform new directions in dementia policy, encouraging systems that value creativity, inclusion and holistic models of support.

Delacourt et al. contribute an ‘Original Research' article describing a Canadian assisted living setting where co-viewing emotionally positive videos with people with advanced dementia elicits emotional communication with caregivers, offering social connection and joy in a shared experience. They describe a variety of vocalizations, verbal productions, gaze, and facial expressions as part of emotional communication with caregivers that help to support social engagement and promote wellbeing.

In a “Systematic Review,” Huang et al. examine the evidence on the use of headphones in music programs for people living with dementia within long-term care settings. Their work identifies multiple enablers and barriers to implementing these programs for people living with dementia in these settings, which can further support the use of personalized music-based therapy with people living with dementia.

In a “Perspective” article, an international group of authors led by Liougas et al. that included people with lived experience describe their experience of shifting the dementia narrative by participating in Walking the Talk for Dementia, a global immersive multi-day event that brings together people with lived experience of dementia, researchers, advocates, clinicians, and artists. Their work highlights that when people living with dementia and their care partners are genuinely included and valued, dementia narratives shift from deficit-focused models to one that recognizes creativity, resilience, love, and shared humanity as foundations for dignity, agency, and meaningful connection.

In another “Perspective” article, O'Kelly et al. describe an art-neuroscience initiative that transforms short segments of a person's EEG brain activity into colored spectrograms, which are then rendered as stained glass and installed in the person's own home. By using light, color, and brain-derived imagery to create dynamic and immersive spaces, the article offers a creative approach to dementia care that shifts attention toward presence, sensory experience, and patient-centered collaboration.

## 5 Policy to support people living with dementia

Innovations in the care and support of people living with dementia and their care partners is essential as Angelopoulou et al. address in their “Original Research” article which validates an assessment of caregiver distress. However, the need to better support people living with dementia along with their care partners is increasingly recognized as a policy priority across the globe (Dawson and Comas-Herrera, 2022; Kim et al., 2026). While some countries may have policies and programs already in place, these are often inadequate in scope and scale to create equitable and capable care systems, as described by Boughrum et al. in their “Opinion” article. Other countries may lack the infrastructure or are under-resourced, making the implementation of supports that can allow individuals and care partners to thrive difficult. Novel approaches to policy development and implementation, highlighted in several manuscripts in this Research Topic, begin to address existing gaps in support by sharing best practices in dementia policy.

Roberts et al. report on the adaptation of the Alzheimer's and Dementia Care ECHO Program and its potential impact on public health professional practice and knowledge change regarding dementia risk reduction in an “Original Research” article. The findings suggest that increased recognition of and confidence to implement dementia risk reduction strategies can lead to improved health outcomes and more proactive measures being integrated into local and regional public health strategies.

Nakakawa et al. further illuminate how dementia policy is translated into everyday homecare practice in Sweden, showing that person-centered care requires more than formal policy commitments in their “Original Research” article. By using participatory, design-driven methods to create spaces for collective sensemaking across system levels, the study offers an innovative approach to bridging the gap between policy ideals and the realities of municipal dementia care.

In their “Brief Research Report,” Carder et al. and a US-based team examined how different states incorporate person-centered care and family-involved care in their policies and regulations for assisted living. US states with memory-care-specific assisted living regulations were far more likely to include detailed person-centered and family-involved care requirements, highlighting key policy areas such as staff training, care planning, resident rights, and social engagement that may influence health outcomes for people living with dementia.

In a “Perspective” article, Snowball et al. in Canada examine how people living with dementia and care partners are involved in shaping research, policy and advocacy across four national and international network contexts. Drawing on practical examples, the article highlights shared challenges and opportunities in meaningful involvement and argues that lived experience should be recognized as a form of expertise, supported through sustained participation, accessible infrastructures, diversity and inclusion, evaluation of impact, and international collaboration.

## 6 Technology for dementia care and support

Technological innovation is increasingly recognized as an important component of dementia care and support, offering opportunities to enhance independence, facilitate social participation, expand access to services, and facilitate support for family carers (Almeida et al., 2026; Pappadà et al., 2021). However, technology alone is not enough; its success depends on how well it serves the needs, preferences and lived experience of people living with dementia and their supporters. The articles under this theme explore both the promise and practical realities of technology-enabled dementia care and support, highlighting the importance of co-design, effective implementation and person-centered approaches.

Tsabary et al. report the findings of an INTERDEM study in an “Original Research” article that mapped 28 recent research initiatives involving digital technology use by people with dementia during the COVID-19 pandemic. Drawing on this work, the authors developed a practical, consensus-based framework to guide the adoption and implementation of assistive technologies, providing valuable direction for researchers, practitioners and policymakers seeking to maximize the benefits of technology in dementia care.

In a “Review” article published in *Frontiers of Public Health* and included in this Research Topic, Szcześniak et al. further advance how assistive technologies can support social health in dementia, including independence, participation, and meaningful engagement. The review is innovative in shifting attention from technology availability alone to equitable implementation, highlighting the need for personalisable, usable, diversity-sensitive technologies.

In an “Opinion” piece, Kafadar et al. examine the opportunities and limitations of online peer support for people affected by young-onset dementia. The paper emphasizes the importance of equitable and age-appropriate support, particularly for underserved populations facing barriers related to geography, digital access, stigma and service availability, while calling for more inclusive and co-designed approaches to online dementia support.

In a “Perspective” article from Spain and The Netherlands, Vilor-Tejedor et al. recognize the potential of Artificial Intelligence to enhance care delivery, support decision-making and improve efficiency. However, the authors argue that the development and governance of AI must be grounded in principles of dignity, equity, privacy and human connection, ensuring that technology augments rather than replaces human care.

In their “Original Research” article, Timmons et al. describe the implementation of an online education program and demonstrate how peer support elements embedded within the program helped reduce isolation, increase confidence and facilitate knowledge sharing among caregivers. Their findings highlight the potential of online interventions to extend access to education and support while fostering connection among those providing care.

In their “Perspective,” Loseto-Gerritzen et al., seek to illustrate the way that technology, and artificial intelligence (AI) in particular, may be used to support person-centered, multidisciplinary dementia care. The article offers several examples of how this might be implemented within dementia care.

## 7 Conclusion

Innovation is essential to meet the growing need associated with dementia across the globe. What becomes clear within this Research Topic is that numerous individuals and organizations worldwide are developing innovative approaches to better support people living with dementia and their care partners. These innovations transcend traditional services and support for people living with dementia highlighting new approaches, as well as innovations in national or regional policies that can be transferable across jurisdictions. The diversity of articles and topics presented in this Research Topic invites readers to explore this work in greater detail and to consider how they might include some of these concepts in their own ongoing work in dementia.
